# CD63 Immunological Activation Versus Hemostatic Function: Platelet Alterations After Polytrauma

**DOI:** 10.3390/cimb47070510

**Published:** 2025-07-02

**Authors:** Gregor Roemmermann, Olivia Bohe, Laura Heimann, Franziska Wirth, Franziska Drumm, Peter Biberthaler, Philipp Moog, Christina Schwenk, Nadja Muehlhaupt, Li Wan, Marc Hanschen

**Affiliations:** 1 Department of Trauma Surgery, Klinikum Rechts der Isar, TUM University Hospital, School of Medicine, Technical University of Munich, 81675 Munich, Germany; gregor.roemmermann@mri.tum.de (G.R.);; 2 Experimental Trauma Surgery, Klinikum Rechts der Isar, TUM University Hospital, School of Medicine, Technical University of Munich, 81675 Munich, Germanyli.wan@tum.de (L.W.); 3 Department of Plastic Surgery and Hand Surgery, Klinikum Rechts der Isar, TUM University Hospital, School of Medicine, Technical University of Munich, 81675 Munich, Germany

**Keywords:** polytrauma, immune response, platelet function, platelet activation, CD63, hemostasis, post-traumatic coagulation disorders

## Abstract

Platelets are attributed an increasing role in the post-traumatic immune response. The exact mechanisms, particularly the link between immune response and hemostasis, have not been conclusively established. This study aimed to investigate the activity marker CD63 on platelets after polytrauma and its significance for hemostasis. A non-interventional, prospective clinical study was conducted, in which the blood of 20 polytraumatized patients was analyzed at nine time points within 10 days following trauma. Peripheral blood platelets were analyzed using flow cytometry to determine CD63 expression and rotational thromboelastometry (ROTEM^®^) for hemostatic platelet function. Additionally, the clinical parameters of age, gender, and injury severity were correlated to the experimental outcomes. During the observation period, an increase in platelet count and CD63 expression was observed. Simultaneously, a hemostasiological dysfunction with reduced platelet maximum clot firmness (MCF) was observed. The factors of age, gender, and injury severity showed no significant influence on immunological activation or coagulation function. These results suggest that polytrauma induces a platelet response and CD63 activation while simultaneously impairing hemostasis. This reveals a novel perspective on post-traumatic coagulation disorders, indicating that immunologically active platelets may lose their ability to contribute effectively to blood clotting. Consequently, these findings emphasize the critical role of platelet immunology in hemostatic regulation.

## 1. Introduction

Severe trauma is still the leading cause of death in industrialized countries in patients under the age of 45. In its “Injury and Fatality Report”, the World Health Organization stated that, worldwide, the number of deaths from unintentional injuries was approximately three times higher than from HIV, tuberculosis, and malaria combined [1].

The classic trimodal model of trauma mortality was described by Trunkey and coworkers as early as 1983 [2]. In this model, 50–60% of the patients who die after trauma die within the first few minutes (immediate death). They therefore do not survive the so-called “first hit”, mostly a combination of the trauma itself and concomitant tissue disruption. The second peak of death occurs within the first 24 h after trauma and is considered an early death. Since early deaths regularly occur inside the hospital, they can possibly be reduced by optimal early trauma care. However, studies have shown that the proportion of early deaths following trauma has remained unchanged in recent years [3]. The third group, that of late deaths, was originally classified as deaths that occurred more than one week after trauma, but more recent studies also include deaths happening after the first 24 h after trauma. Even though these late deaths could have been significantly reduced over the last decades, they still make up 10–20% of trauma deaths [4,5]. The main causes of late deaths are sepsis and multiple organ dysfunction syndrome, leading to multiple organ failure (MOF) [6]. An important role in those cascades is played by post-traumatic coagulation disorders.

Trauma-induced coagulopathy, influenced by platelet function, is a critical part of the “lethal triad” in traumatic shock, alongside hypothermia and acidosis. Studies indicate that coagulopathy increases mortality risk significantly after trauma, underscoring the platelets’ influence on both immediate and long-term survival outcomes [7,8]. Standard tests, such as the prothrombin time or the activated partial thromboplastin time, provide important information and are part of routine clinical blood tests. However, a comprehensive assessment of coagulation dynamics, including information on clot formation, stability, and fibrinolysis, can only be carried out using more comprehensive testing methods such as thromboelastometry.

Following severe trauma, the innate immune system is rapidly activated. Circulating leukocytes, particularly neutrophils and monocytes, are rapidly mobilized and contribute to the early inflammatory response by releasing pro-inflammatory cytokines such as TNF-α, IL-6, and IL-1β [9]. This immediate immune response plays a critical role in pathogen defense and tissue repair. However, an excessive or unbalanced activation may also promote systemic inflammation and contribute to post-traumatic complications. While the first hit normally leads to a limited activation of the systemic inflammatory response syndrome (SIRS) and the concomitant compensatory anti-inflammatory response syndrome (CARS), subsequent second hits can lead to excessive immune reactions and immunological imbalances. Typically, second hits are caused by operations, hypoxia, cardiovascular instability, and infections [10,11]. The immunologic reaction to the second hit consists of complex interactions between pro- and anti-inflammatory immune cells. Due to immune conditioning from previous exposure, exaggerated immune responses are more likely upon subsequent exposure. This imbalance of SIRS and CARS increases the susceptibility to organ failure or systemic infection. At present, the body’s immune response to trauma is not fully understood. There are no standardized methods to precisely monitor these immune reactions, nor are there established interventions targeting the immunologic pathways. Since the first hit in trauma can almost never be altered, focusing on second hits and the resulting immunologic reaction is essential for improving trauma care and reducing mortality in this field.

Therefore, investigating the molecular pathways has received increasing attention in recent years. Platelets have recently been ascribed an increasingly important role as immune mediators. Since it was discovered that platelets express all nine Toll-like receptors (TLRs) and other signaling surface proteins, like the tetraspanin family, it has been clear that platelets not only play a central role in hemostasis, but also work as active agents within the immune system. Upon activation, platelets release various pro-inflammatory cytokines, including IL-1β, TNF-α, IL-1, and IL-6, along with lipid mediators. These TLRs enable the platelets to recognize pathogen-associated molecular patterns, allowing them to bind and neutralize antigens either through encapsulation or by releasing antimicrobial peptides [12]. In addition, platelets communicate with and influence other immune cells.

Following severe trauma, platelets assume a dual role. In addition to their primary function in clotting, they release both pro- and anti-inflammatory mediators, which significantly impact the immune environment [13]. Preliminary studies by our research group have shown that platelet activity can directly influence CD4+ regulatory T cells (T_reg_) [14]. An imbalance in these responses may lead to complications such as sepsis or MOF.

One of the less extensively studied surface markers on platelets is the tetraspanin CD63. It is expressed on various cell types, including immune cells such as mast cells, dendritic cells, T_regs_, and platelets. By clustering with other cell surface receptors, CD63 is involved in multiple physiological pathways of cell signaling, adhesion, and molecular trafficking. After trauma, CD63 can modulate inflammatory responses by regulating the activation, migration, and proliferation of immune cells to the injured tissue. Those processes are realized through the release of inflammatory markers and cytokines, such as TNF-alpha [15].

On platelets, CD63 is located in the endosomal system of the alpha granula and on the cell surface [16]. The surface expression is increased with platelet activation, enabling CD63 to be used as an activity marker of platelets [17]. CD63 is involved in the signaling pathways that mediate platelet activation and contribute to their amplification process [18]. However, the full extent of the involvement of the tetraspanin family after trauma is not fully understood, highlighting the need for further research in this field. Understanding the immunological activation of platelets and their concomitant hemostatic dysfunction in the setting of severe trauma is crucial for improving therapeutic strategies.

We hypothesized that the activation level of CD63 transmitters increases significantly after trauma and, at the same time, increases the activation and hemostatic power of platelets.

## 2. Materials and Methods

### 2.1. Study Population and Sampling Protocol

In this prospective, non-interventional trial, 20 severely injured patients were enrolled at the trauma room of a German Level I trauma center between February and September 2021. Enrollment was based on clinical presentation, injury pattern, and the need for interdisciplinary trauma care. Final study inclusion required a retrospectively calculated Injury Severity Score (ISS) of ≥16, in accordance with established criteria for severe trauma used by the TraumaRegister DGU^®^. Written informed consent was obtained from all patients or their legal representatives.

Patients were excluded when the time from trauma to presentation in the ER exceeded 12 h or when patients were under 18 years of age. Also, secondary transfers from other hospitals were excluded. Further exclusion criteria were pregnancy and incarceration. Enrollment also depended on the availability of the study team and capacity in the research laboratory. Pre-existing antithrombotic medication was not defined as an exclusion criterion, as reliable medication histories are often not available in acute emergency settings involving severely injured patients.

Nine sequential blood samples were taken and analyzed: in the trauma room (with a maximum of 60 min post-admission); at 6, 12, 24, 48, and 72 h; and 5, 7, and 10 days after trauma. The trauma-room samples were obtained prior to any transfusion. Blood was drawn and collected in commercially available monovettes (Sarstedt AG & Co., Nümbrecht, Germany) either laced with ethylenediaminetetraacetic acid for flow cytometry or citrate for thromboelastometry.

Clinical data on patients, such as age, gender, trauma mechanism, and the exact injury pattern with the severity of the injuries, classified according to the AIS, were collected. Additionally, the ISS was calculated. Data on the course of treatment, including the number of surgical interventions, time spent in the ICU, and mortality, were also noted.

### 2.2. CD63 Expression on Platelets

The MACSQuant^®^ 9 Analyzer from Miltenyi Biotec (Bergisch Gladbach, Germany) was used to determine the surface expression markers of platelets. The platelets were isolated, fixed, stained, and then measured by flow cytometry. For sample collection, the patients’ blood was drawn using the standard venipuncture technique or by laying central venous catheters and collected in a 2.9 mL Sarstedt S-Monovette^®^ with 3.2% citrate (Sarstedt AG & Co. KG, Nümbrecht, Germany). First, 1.5 mL of the blood sample was transferred to a 2 mL SafeSeal reaction tube (Sarstedt AG & Co. KG, Nümbrecht, Germany) and centrifuged at 500× *g* and 21 °C for 10 min (Centrifuge 5415 R, Eppendorf SE, Hamburg, Germany) in an inclined suspension. The platelet-rich plasma (PRP) formed at the boundary zone between the centrifugate and serum was then pipetted off and transferred to another 2 mL SafeSeal reaction tube (Sarstedt AG & Co. KG, Nümbrecht, Germany). After renewed centrifugation at 200× *g* and 21 °C for 10 min (Centrifuge 5415 R, Eppendorf SE, Hamburg, Germany), the supernatant was decanted, and the platelets were mixed with 620 µL of phosphate-buffered saline with 0.3% paraformaldehyde (PFA 0.3%, see Table A1) for fixation. In each case, 100 µL was distributed in 6 wells of a 96-well round-bottom plate (Sarstedt AG & Co. KG, Nümbrecht, Germany) and incubated at room temperature under light protection for 30 min.

Meanwhile, the antibody mixture was prepared for staining. CD41 (anti-human, HIP8, PacificBlue^TM^, BioLegend, San Diego, CA, USA) was used for identification, and CD63 (anti-human, H5C6, PE, BioLegend, San Diego, CA, USA), CD62P (anti-human, Psel.KO2.3, APC, eBioscience, San Diego, CA, USA), and TLR9 (anti-human, 5G5, FITC, abcam, Cambridge, UK) were used as activity markers and mixed together under light protection. The inclusion of CD62P and TLR9 antibodies in the staining panel reflects a standard protocol for platelet immunophenotyping, which was used by our study group in preliminary studies. These markers were not analyzed in the context of the present study but are reported in Section 2 to ensure full transparency regarding the composition of the antibody mixture.

A total of 1.5 µL of each antibody was used, and the mixture was diluted with 60 µL of phosphate-buffered saline (PBS, see Table A1), resulting in 66 µL of antibody mixture. With an additional 3 wells, each containing approximately 3 mL of platelets to be stained, the recommended antibody dosage of 1:50 was obtained in the test sample.

Once fixation was complete, the platelets were washed with 100 µL PBS, and the round-bottom plate was centrifuged at 500× *g* and 4 °C for 10 min (Centrifuge 5810 R, Eppendorf SE, Hamburg, Germany). The supernatant was then decanted, and the platelets were stained.

In three wells labeled “Thrombo +”, the platelets were resuspended and stained with 20 µL of the antibody mixture, and in the other three wells, labeled “Thrombo −”, the platelets were resuspended with 20 µL of PBS. Staining and subsequent incubation at 4 °C were carried out under light protection. After 45 min of staining, the staining was terminated by adding 200 µL of phosphate-buffered saline with bovine serum albumin (PBA, see Table A1) per well and centrifugation at 500× *g* and 4 °C for 10 min (Centrifuge 5810 R, Eppendorf SE, Hamburg, Germany). After removal of the supernatant, the samples were carefully resuspended in 100 µL PBS, and the round-bottom plate was stored on a Chill 96 Rack (Miltenyi Biotec, Bergisch Gladbach, Germany). The subsequent measurement of the samples with the MACSQuant^TM^ 9 Analyzer (Miltenyi Biotec, Bergisch Gladbach, Germany) was performed after calibration of the device with MACSQuant^TM^ Calibration Beads (Miltenyi Biotec, Bergisch Gladbach, Germany) according to the manufacturer’s instructions. The voltages listed in Table A2 were determined using preliminary tests and set for measurement. Further settings were a low flow rate, a medium resuspension of the samples before uptake, and a washing step between each measurement. A total of 75 µL per well was measured at a rate of up to 10,000 events per second. This resulted in approximately 3 million recognized events per well. All measurements were performed as triplets. For statistical analyses, the mean values of those triplets were used.

### 2.3. Platelet Function

The blood remaining in the 2.9 mL Sarstedt S-Monovette^®^ with 3.2% citrate (Sarstedt AG & Co. KG, Nümbrecht, Germany) from the flow cytometric measurement of platelets was used to determine platelet function. It should be emphasized that the blood samples were analyzed within 10 min after collection. Platelet activation and hemostatic function were measured using ROTEM^®^ (Tem International GmbH, Munich, Germany). The ROTEM^®^ assay involves the continuous rotation of a pin immersed in the blood sample, allowing for the real-time measurement of clot formation and fibrinolysis parameters. Each blood sample was analyzed using the EX-TEM^®^, IN-TEM^®^, FIB-TEM^®^, and NA-TEM^®^ pathways. The blood and coagulation activators used were warmed to 37 °C before the experiment. In the first step, the starting reagents EX-TEM^®^, IN-TEM^®^, and FIB-TEM^®^ (see Table A3) were mixed with calcium (STAR-TEM^®^, see Table A3) in the prepared measuring cuvettes (Werfen GmbH, Munich, Germany). For the NA-TEM^®^ measurement, only the STAR-TEM^®^ reagent was added to the cuvette. The blood sample was then added, the plunger inserted into the cuvette, and the measurement recording started. The quantity of reagents and blood sample used is automatically determined by the electronic pipette of the ROTEM^®^ delta (Werfen GmbH, Munich, Germany). While additional ROTEM^®^ channels (IN-TEM^®^ and NA-TEM^®^) were also recorded for completeness, the analysis focused exclusively on the comparison of EX-TEM^®^ and FIB-TEM^®^ to isolate the platelet-specific contribution to clot firmness [19].

The ROTEM^®^ parameters of clotting time (CT), clot-formation time, MCF, and lysis index were measured. CT refers to the time it takes for the blood to begin clot formation after the initiation of coagulation. CT is measured from the start of the analysis until a predetermined level of clot firmness is reached, which in the case of ROTEM^®^ analysis is set at 20 mm. A prolonged CT may indicate deficiencies in clotting factors, impaired platelet function, or the presence of anticoagulant medications. Conversely, a shortened CT may suggest hypercoagulability or increased thrombin generation.

MFC represents the maximum strength or firmness of the blood clot formed during the coagulation process. It is determined by the maximum amplitude or elasticity of the clot measured on the ROTEM^®^ curve. This point represents the peak of clot firmness reached during the coagulation process. The higher the MCF value, the stronger and more stable the clot is considered to be.

ROTEM^®^ data were acquired and analyzed using the ROTEM^®^ software (Version LibROTEM 20070213 Rawlog {Default-1000}) provided by the manufacturer. Clot formation curves were generated, and ROTEM^®^ parameters were calculated automatically based on the curve characteristics.

### 2.4. Statistical Analysis

Data were analyzed using GraphPad Prism 8 (version 8.0.2.263, GraphPad Software Inc., Boston, MA, USA) and RStudio IDE (version 2024.12.1+563, Posit PBC, Boston, MA, USA). Patients’ characteristics were described using the mean and standard deviation (SD) for continuous variables. Absolute numbers and relative percentages were used for categorical variables. CD63 expression and platelet function were analyzed using linear mixed-effects model analysis (LMM), since this method accounts for both fixed and random effects in repeated-measures data and is robust to missing values. *p*-values <0.05 were considered statistically significant.

## 3. Results

### 3.1. Demographic Data

From 24 February until 13 September 2021, we were able to include twenty patients in this study. Demographic patient data and outcomes are shown in Table 1. The mean time from trauma to admission in an emergency room (ER) was 60.5 min. The mean age at injury was 49.1 ± 17.8 years, with 16 patients (80.0%) being male. All injuries in the study cohort were classified as blunt trauma. Regarding the trauma mechanism, 14 patients (70.0%) were injured in road traffic accidents (4 pedestrians, 3 by bicycle, 2 by vehicle, and 5 by motorcycle), 5 patients were injured due to falls (3 in falls above 3 m and 2 in low falls), and one injury happened by a severe hit. The mean Injury Severity Score (ISS) was 28.1 ± 10.0.

The most frequently injured body regions were head (85%), skin or external (80%), extremities and pelvis (75%), and thorax (70%). The face and abdomen were less frequently affected, each occurring in 45% of the patients. The most severe injuries were to the head, with a mean Abbreviated Injury Score (AIS) of 3.3, abdomen (mean AIS 3.1), and thorax (mean AIS 3.0).

The majority (85%) of patients required treatment in the intensive care unit (ICU), on average for 14.0 ± 8.0 days. MOF occurred in 40% of patients. After 43.3 ± 56.4 and an average of 5.9 ± 4.2 surgeries, patients were able to leave the primary care hospital. One patient died within the first 30 days after trauma (on day three).

Due to death (*n* = 1) or early transfer to another hospital (*n* = 5), a total of six patients did not complete the full 10-day observation period. Combined with time-point-specific missing data (e.g., due to ongoing surgery or clinical instability), this resulted in an overall missingness of 14.4% of the planned measurements.

### 3.2. Platelet Count

**Figure 1:**
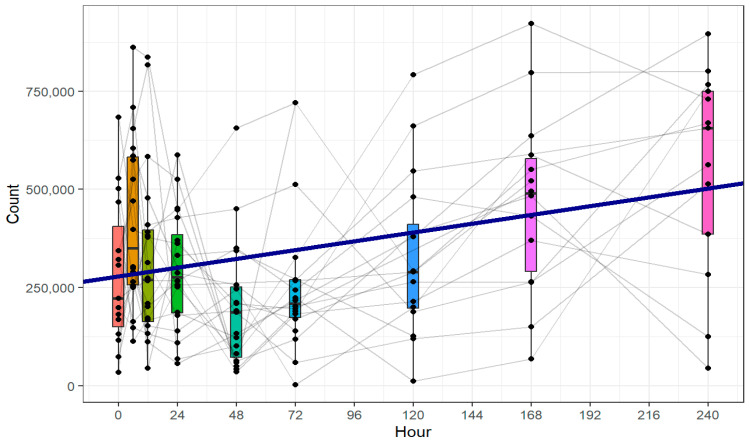
Absolute platelet count in 250 µL of peripheral blood of polytraumatized patients, assessed via flow cytometry. Blood was drawn at nine different time points: in the ER, after 6 and 12 h, and after 1, 2, 3, 5, 7, and 10 days. Each dot represents a single patient and time point. The grey lines represent single patients’ courses. The blue fit line represents the linear mixed-effects model. There was a significant increase in the absolute platelet count during the first 10 days after trauma (+93.8% from ER to day 10, m = 931.59, 95% confidence interval (CI) [505.00; 1358.18], *p* < 0.001, k = 279,098.81, 95% CI [218,247.35; 339,950.26], *p* < 0.001).

Figure 1 shows the absolute number of platelets over the course of the first 10 days after trauma. Platelets were identified using single-cell gating, matching forward scatter (FSC) over side scatter (SSC) configuration, and CD41 positivity. The LMM analysis showed a significant increase in platelet count over time. The included grey lines illustrate individual patient trajectories and were added to reflect the considerable inter-individual variability in response patterns. Although Figure 1 suggests a transient decline in platelet counts at 48 and 72 h, separate statistical analyses of these time points did not reveal any significant differences relative to preceding measurements. As presented in Table 2, the covariates of age, gender, and ISS showed no significant influence on the development of the platelet count.

### 3.3. CD63 Expression on Platelets

**Figure 2:**
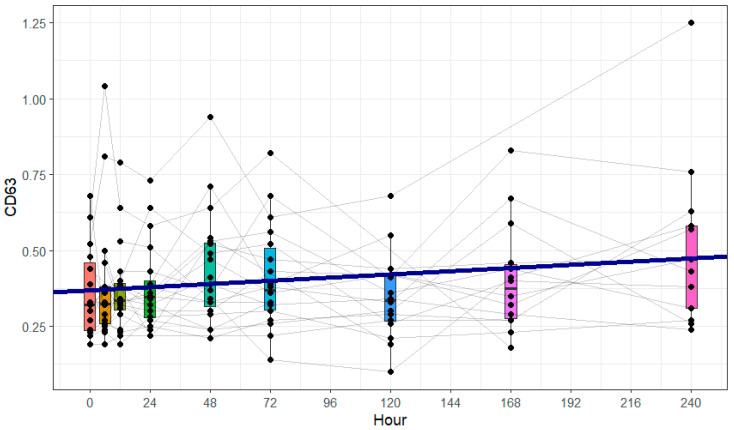
CD63 mean fluorescence intensity (MFI) on platelets in peripheral blood of polytraumatized patients, assessed via flow cytometry. Blood was drawn at nine different time points: in the ER, after 6 and 12 h, and after 1, 2, 3, 5, 7, and 10 days. Each dot represents a single patient and time point. The grey lines represent single patients’ courses. The blue fit line represents the linear mixed-effects model. The change in CD63 was calculated per 24 h. There was a significant increase in the CD63 expression during the first 10 days after trauma (+40.4% from ER to day 10, m = 0.01046, 95% CI [0.00339 to 0.01752], *p* = 0.004, k = 0.39990, 95% CI [0.21086 to 0.58895], *p* < 0.001).

The expression of CD63 on platelets is displayed in Figure 2. Platelets were identified using single-cell gating, matching FSC over SSC configuration, and CD41 positivity. The CD63 expression of the platelets was measured using the antibody CD63 (anti-human, H5C6, PE, BioLegend, San Diego, CA, USA). The mean MFI of the triplet measurement was used for calculating the LMM in RStudio. The model showed a significant increase in the observation period. Subgroup and covariate analyses were performed for the parameters of gender, age, and ISS but revealed no significant influence (see Table 3).

### 3.4. Hemostatic Platelet Function

**Figure 3:**
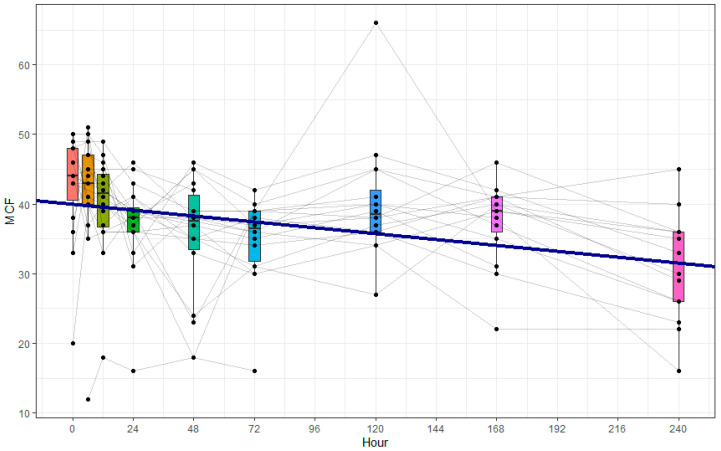
Platelet MCF over the first ten days after trauma in polytraumatized patients over the first 10 days after trauma, calculated by subtraction of FIB-TEM^®^ MCF (representing the extrinsic coagulation pathway with inhibition of platelet coagulation through cytochalasin) from EX-TEM^®^ MCF (representing the extrinsic coagulation pathway), both assessed via ROTEM^®^ analysis. Blood was drawn at nine different time points: in the ER, after 6 and 12 h, and after 1, 2, 3, 5, 7, and 10 days. Each dot represents a single patient and time point. The grey lines represent single patients’ courses. The blue fit line represents the linear mixed-effects model. There was a significant decrease in the platelet MCF during the first 10 days after trauma (−27.1% from ER to day 10, m = −0.03516, 95% CI [−0.04878 to −0.02154], *p* < 0.001, k = 40.01503, 95% CI [37.29247 to 42.73758], *p* < 0.001).

The overall clotting function measured by the maximum clot firmness of EX-TEM^®^, IN-TEM^®^, and FIB-TEM^®^ channels in ROTEM^®^ increased over the 10 days after trauma, whereas the platelet-specific clotting function represented by the subtraction of FIB-TEM from EX-TEM MCF significantly decreased in the observed period. The LMM calculation is shown in Figure 3. The subgroup and covariate analyses for age, gender, and ISS showed no significant influences (see Table 4). To further investigate the potential influence of trauma severity, patients were stratified post hoc into two subgroups based on the median ISS (ISS < 33 vs. ≥33). No statistically significant differences in CD63 expression or hemostatic platelet function were observed between the subgroups.

## 4. Discussion

Trauma-induced coagulopathy (TIC) is a well-documented phenomenon, characterized by a complex interplay among systemic inflammation, endothelial dysfunction, and platelet alterations [7]. Coagulopathies are of particular relevance in the context of polytrauma, as they are associated with an increased need for blood products, higher rates of organ failure, and elevated overall mortality [20]. The mean ISS in our study cohort was 28.1, with most of our patients suffering from multiple injuries. Head and chest injuries, which were among the most frequent injuries, are known to have significant effects on systemic inflammation and coagulation pathways [21]. The high prevalence of extremity and pelvic injuries may further contribute to thromboinflammatory processes due to extensive soft tissue damage and prolonged immobilization. The observed study population is therefore well-suited to conduct observations on post-traumatic platelet activation and TIC.

One central observation of our study was that increased CD63 expression, indicating immunological platelet activation, was paralleled by a decrease in clot firmness attributable to platelets, as assessed by ROTEM^®^. Furthermore, we observed a significant increase in the absolute platelet count.

Flow cytometry was used not only to determine platelet numbers but, more importantly, to assess immunological activation at the single-cell level. This method allowed us to directly evaluate the surface expression of CD63 on platelet populations, which would not have been possible using standard hematology analyzers. Although flow cytometric platelet counting may be affected by sample preparation, its ability to provide detailed functional phenotyping made it the method of choice for this study. We acknowledge that the EX-TEM^®^ minus FIB-TEM^®^ MCF difference is only an indirect estimate of platelet function and is subject to several confounding factors. It lacks diagnostic specificity and is not established as an outcome predictor. In a recent study, global coagulation parameters such as clotting times remained within normal ranges, while platelet dysfunction was clearly detectable only through targeted assessments. These findings emphasize the need for platelet-specific testing, such as the ROTEM^®^-based differential approach used in our study, to accurately detect functional impairments after trauma [22].

CD63 is a well-established marker of platelet activation. It is translocated to the platelet surface upon α-granule release and has been widely used in flow cytometric studies to quantify platelet degranulation. Its expression correlates with activation status in both experimental and clinical settings, and it plays a role in tetraspanin–integrin signaling complexes [23,24,25]. Elevated CD63 expression after trauma indicates increased immunological platelet activation, consistent with the known systemic inflammatory response and thromboinflammatory processes [17,26]. This observation is in agreement with previous studies showing that trauma induces a hyperactive platelet phenotype [27]. However, concurrent with the immunological activation, we observed a decrease in platelet coagulation function. The hypothesis of increased platelet coagulation activity after severe trauma must therefore be rejected. Our results emphasize the need to investigate the different platelet functions together in future studies in order to gain a more comprehensive understanding of post-traumatic platelet dysfunction.

Trauma-induced platelet dysfunction has been extensively described in the literature [7,28,29,30]. However, the exact mechanisms responsible for the observed decline in platelet hemostatic function remain unclear. Potential contributors include direct platelet exhaustion, interactions with leukocytes and the endothelium, and alterations in platelet receptor signaling [31,32,33]. In this context, recent findings suggest that the trauma-induced loss of platelet surface receptors, particularly GPVI and GPIbα, may play a critical role in reducing platelet responsiveness during ongoing bleeding [34]. Additionally, transcriptomic studies have identified trauma-associated changes in alternative RNA splicing within platelets, suggesting a possible molecular basis for impaired platelet function and TIC [35]. Our findings expand on these observations by indicating a possible dissociation between immunological activation, reflected by increased CD63 expression, and hemostatic capacity, as measured by clot firmness.

An alternative explanation for the observed reduction in clot firmness could be platelet exhaustion, a phenomenon in which sustained activation leads to a functional decline over time. This concept has been described in inflammatory and thromboinflammatory settings, where hyperactivated platelets progressively lose their hemostatic competence despite ongoing stimulation [30,36,37]. However, in our study, the decline in ROTEM^®^-based platelet-dependent clot firmness occurred in parallel with the early increase in CD63 expression, not as a delayed consequence. This temporal pattern suggests that functional impairment and immunological activation may occur simultaneously rather than sequentially and may reflect distinct regulatory mechanisms following trauma.

In recent years, the interplay between inflammation and thrombosis has garnered increasing attention under the concepts of “thromboinflammation” and “immunothrombosis”. However, the precise underlying mechanisms remain an active area of research, and there is a lack of pharmacological approaches to address the observed complications effectively [38]. To the best of our knowledge, this is the first prospective study to simultaneously examine both the immunological activation of platelets and their hemostatic capacity in severely injured patients. Previous research has either focused on platelet activation markers or functional deficits separately.

Our data indicate that patient age, sex, and ISS had no significant influence on platelet activation or function. This finding suggests that trauma severity alone does not dictate platelet behavior and that other factors, such as immune status, immunological priming, or endothelial interactions, may play a more critical role. This contrasts with some prior reports suggesting that age and sex influence coagulation responses post-trauma, warranting further investigation into potential underlying mechanisms [39,40].

The nature of this prospective, non-interventional study imposes limitations on the scope and interpretability of its findings.

The study population of 20 patients is relatively small, especially for conducting subgroup analyses. This limitation results from the study’s complex design and the rarity of the target population: patients with severe trauma. Subgroup analyses resulted in cohorts of partly fewer than 10 patients, rendering the absence of statistically significant differences in CD63 expression and platelet hemostatic function likely attributable to insufficient statistical power and data variability. To overcome this problem, patients’ characteristics were analyzed as covariables in the LMM. The LMM offers significant advantages in handling missing data, accounting for individual variability through random effects, and modeling within-subject correlations. Unlike traditional methods, it provides robust statistical power and allows for the inclusion of covariates, making it a valuable tool for analyzing complex longitudinal data, especially when the focus is on overall trends rather than specific time points, as pointed out in our study. However, the use of LMM for statistical analyses has limitations, as it does not compare discrete time points directly or conduct analyses on an individual-patient level. Instead, the LMM identifies overall trends across the dataset, often assuming linear change over time. Consequently, transient or non-linear temporal dynamics may not be accurately detected.

An additional limitation lies in the potential influence of pre-injury antithrombotic medication, which could not be systematically excluded. In the acute emergency setting of severe trauma, reliable medication histories are often unavailable at the time of admission, and pre-existing antithrombotic use may therefore have introduced variability into the observed platelet phenotypes. Additionally, while no patients received blood products prior to the initial blood sampling, several required transfusions during the subsequent course of treatment. These interventions may have influenced later platelet phenotypes and ROTEM^®^ measurements and represent a potential confounding factor.

We acknowledge that ROTEM^®^ is not a direct measure of platelet function in the classical sense. However, the differential approach using EX-TEM^®^ and FIB-TEM^®^ is well established in trauma research and allows for a valid approximation of the platelet contribution to clot firmness. While not providing mechanistic insights into platelet signaling, this method enables the objective tracking of temporal changes in platelet-dependent coagulation activity.

A formal correlation analysis between CD63 expression and platelet-specific clot firmness (MCF) was not performed in this study. As an exploratory pilot study in a rare and critically injured patient population, our primary aim was to describe temporal changes in immunological and hemostatic platelet behavior rather than to test predefined mechanistic hypotheses. The observed divergence between CD63 upregulation and declining MCF represents a hypothesis-generating finding that could inform the design of future studies. Investigating potential mechanistic links, such as direct correlation or causal relationships, between platelet activation and functional impairment will require larger, targeted cohorts with broader analytical designs.

Nonetheless, the primary objective of this pilot study was to explore cellular mechanisms following polytrauma. The study design is well-suited for this exploratory purpose and serves as a starting point for future research projects. This publication aims to offer initial insights and lay the groundwork for future studies, rather than establishing causal or mechanistic relationships.

## 5. Conclusions

Our study provides novel insights into the immunological activation and hemostatic dysfunction of platelets following severe trauma.

Overall, we demonstrated an increase in CD63 expression in platelets within the first 10 days following severe trauma, accompanied by a simultaneous decrease in the hemostatic function. The occurrence of coagulation dysfunction alongside heightened immunological platelet activation suggests a potential correlation between immunological activation and the loss of hemostatic platelet functionality in the context of polytrauma.

This publication highlights the immunological activation of platelets as a potential underlying mechanism for the development of post-traumatic coagulation disorders. In doing so, it provides a significant contribution to the future development of post-traumatic immunomodulatory therapies and potentially the prevention of coagulopathic complications.

## Figures and Tables

**Table 1:** Demographic patient data and outcomes.

	Number (Percentage)	Mean ± Range
Patient Characteristics		
Number of Patients	30	
Study patients	20 (100%)	
Age (in years)		51.9 ± 17.4
Sex		
Male	16 (80%)	
Female	4 (20%)	
Trauma Mechanism		
Traffic accidents	14 (70%)	
Falls	5 (25%)	
Other	1 (5%)	
Injury Pattern		
Glasgow Coma Scale on scene/admission		12.0 ± 4.0/10.2 ± 5.3
Head or neck injury, AIS	17 (85%)	3.3 ± 1.4
Face injury, AIS	9 (45%)	1.9 ± 0.6
Chest injury, AIS	14 (70%)	3.0 ± 0.9
Abdomen or pelvis content injury, AIS	9 (45%)	3.1 ± 0.6
Extremities or pelvic girdle injury, AIS	15 (75%)	2.9 ± 0.6
Skin or external injury, AIS	16 (80%)	1.5 ± 0.5
ISS		28.1 ± 10.0
Treatment and Outcome		
Length of Stay		
ICU (in days)	17 (85%)	14.0 ± 14.7
In-hospital (in days)		43.3 ± 56.4
Treatment		
Patients receiving surgery, N° of surgeries	17 (85%)	5.9 ± 4.2
MOF	8 (40%)	
Mortality	1 (5%)	
Time to death (days)		5
Patients Not Completing the Full Observation Period		
Due to early transfer	5 (25%)	
Due to death	1 (5%)	

**Table 2:** Covariates LMM analysis for absolute platelet count; gender was set to male = 1, female = 2, and age and ISS were used as linear variables.

Covariate	Estimate (m)	95% CI	*p*-Value	*p*-Rating
Age	2113.92	−976.26 to 5204.09	0.178	NS ^1^
Gender	−95,763.10	−224,275.35 to 32,749.15	0.143	NS ^1^
ISS	−1742.73	−7174.18 to 3688.72	0.527	NS ^1^

^1^ NS: not significant.

**Table 3:** Covariates LMM analysis for CD63 expression on platelets; gender was set to male = 1, female = 2, and age and ISS were used as linear variables.

Covariate	Estimate (m)	95% CI	*p*-Value	*p*-Rating
Age	0.00100	−0.00245 to 0.00445	0.567	NS ^1^
Gender	−0.02648	−0.17509 to 0.12212	0.725	NS ^1^
ISS	0.00054	−0.00544 to 0.00652	0.859	NS ^1^

^1^ NS: not significant.

**Table 4:** Covariates LMM analysis for platelet MCF; gender was set to male = 1, female = 2, and age and ISS were used as linear variables.

Covariate	Estimate (m)	95% CI	*p*-Value	*p*-Rating
Age	−0.00414	−0.01025 to 0.00197	0.182	NS ^1^
Gender	−0.03437	−0.30743 to 0.23870	0.804	NS ^1^
ISS	−0.00647	−0.01702 to 0.00408	0.228	NS ^1^

^1^ NS: not significant.

## Data Availability

The data obtained is available on reasonable request from the authors.

## Abbreviations

The following abbreviations are used in this manuscript:

MCF	Maximum Clot Firmness
MOF	Multiple Organ Failure
SIRS	Systemic Inflammatory Response Syndrome
CARS	Compensatory Anti-Inflammatory Response Syndrome
TLR	Toll-Like Receptor
T_reg_	Regulatory T Cell
ER	Emergency Room
ISS	Injury Severity Score
AIS	Abbreviated Injury Score
ICU	Intensive Care Unit
FSC	Forward Scatter
SSC	Side Scatter
LMM	Linear Mixed-Effects Model
MFI	Mean Fluorescence Intensity
CI	Confidence Interval
TIC	Trauma-Induced Coagulopathy
PFA 0.3%	Phosphate-Buffered Saline with 0.3% Paraformaldehyde
PBS	Phosphate-Buffered Saline
PBA	Phosphate-Buffered Saline with Bovine Serum Albumin
CT	Clotting Time

## Appendix A

**Table A1:** Solutions used in platelet preparation.

Ingredient	Quantity
PBS	1 L
PBS solution with MgCl/CaCl ^1^	1 L
PBA	1 L
PBS solution with MgCl/CaCl ^1^	1 L
Bovine serum albumin ^1^	10 g
Sodium azide ^1^	1 g
PFA 0.3%	500 mL
PBS solution with MgCl/CaCl ^1^	496 mL
Formaldehyde 37% ^1^	4 mL

^1^ Manufactured by Sigma-Aldrich, Merck KGaA, Darmstadt, Germany.

**Table A2:** Voltages used for flow cytometric platelet measurement.

Channel	FSC	SSC	B1	B2	B3	B4	V1	R1	R2
Voltage	272	462	402	402	416	472	378	564	496

**Table A3:** Coagulation activators used for ROTEM^®^.

	Components	Coagulation System
EX-TEM^® 1^	Tissue thromboplastin	Extrinsic pathway
IN-TEM^® 1^	Ellagic acid	Intrinsic pathway
FIB-TEM^® 1^	Tissue thromboplastin and cytochalasin D	Extrinsic pathway and platelet inactivation
STAR-TEM^® 1^	Calcium	Non-activated coagulation

^1^ Manufactured by Werfen GmbH, Munich, Germany.
